# Real-Time Maritime Traffic Anomaly Detection Based on Sensors and History Data Embedding

**DOI:** 10.3390/s19173782

**Published:** 2019-08-31

**Authors:** Julius Venskus, Povilas Treigys, Jolita Bernatavičienė, Gintautas Tamulevičius, Viktor Medvedev

**Affiliations:** Institute of Data Science and Digital Technologies, Vilnius University, Akademijos str. 4, LT-08412 Vilnius, Lithuania

**Keywords:** streaming sensors data, neural network retrain time, model sensitivity and precision, marine traffic anomaly detection, SOM data batching

## Abstract

The automated identification system of vessel movements receives a huge amount of multivariate, heterogeneous sensor data, which should be analyzed to make a proper and timely decision on vessel movements. The large number of vessels makes it difficult and time-consuming to detect abnormalities, thus rapid response algorithms should be developed for a decision support system to identify abnormal movements of vessels in areas of heavy traffic. This paper extends the previous study on a self-organizing map application for processing of sensor stream data received by the maritime automated identification system. The more data about the vessel’s movement is registered and submitted to the algorithm, the higher the accuracy of the algorithm should be. However, the task cannot be guaranteed without using an effective retraining strategy with respect to precision and data processing time. In addition, retraining ensures the integration of the latest vessel movement data, which reflects the actual conditions and context. With a view to maintaining the quality of the results of the algorithm, data batching strategies for the neural network retraining to detect anomalies in streaming maritime traffic data were investigated. The effectiveness of strategies in terms of modeling precision and the data processing time were estimated on real sensor data. The obtained results show that the neural network retraining time can be shortened by half while the sensitivity and precision only change slightly.

## 1. Introduction

The maritime industry is an important part of the global trade system with a growing volume, intensity, and needs. In 2018, 1.9 billion tons of goods were transported as part of EU short sea shipping [1]. This is 3.2% more in comparison with 2016. Totally, more than 90% of cargo is carried by sea transport [2].

Such growth presents some challenges in the industry. Increasing intensity of maritime traffic raises the need for incident prevention-oriented traffic control. The maritime anomaly or abnormal movement detection is one of the control techniques. It is based on vessel trajectory analysis and search of irregular, illegal, and other anomalous appearances in trajectory data [3]. A maritime trajectory can include vessel identification data, traffic parameters (e.g. speed and rotation), auxiliary data (e.g., meteorological data) for a vessel, and such dataset presents a large-scale, complex data structure. Automated data gathering systems (e.g., Automatic Identification System) return larger and larger trajectory datasets, which are challenging for human-based analysis and anomaly detection [4]. Nowadays, machine learning-based data analysis and mining techniques is a natural choice for this type of task: the obtained structure of data, the extracted information, detected data regularities could help to estimate vessel movement and make some safety decision, to enable the automatic anomaly detection even. For real-world applications, a challenge of real-time operation, data generalization arises. Movement anomalies are detected as history-based deviations of vessel’s trajectory data, which can be problematic considering massive trajectory data streams. In this case, constant estimation of historical and context data means permanent need for system retraining. Full retraining is a time- and power-consuming process; therefore, some techniques of additional or adaptive training would be preferred: rapid self-learning algorithms have to be developed to detect the abnormal movement in stream data.

The paper is organized as follows. Section 2 presents the problem of abnormal movement detection in maritime traffic data and gives the state-of-the-art problem solutions. In Section 3, the motivation of this paper is presented: two retraining strategies are introduced for neural network-based real-time maritime anomaly detection. The results of experimental research of these strategies are given in Section 4. The investigation results are concluded in Section 5.

## 2. Review

In this section, we present maritime anomaly detection task and review some recent research results in this area.

The abnormal vessel movement can be defined as an unreasoned movement deviation from the sea lanes, trajectory, speed or other traffic parameters [5]. As most vessels have the Automated Identification System (AIS) installed, giving the static and dynamic information about the vessel movement, the detection of traffic anomaly comes as the task of data analysis and outlier detection. In addition, different sensor systems can be connected to the AIS. Traffic data are analyzed in point-based or trajectory-based manner [6].

In the first case, every single data point (message from the vessel to the AIS) or a group of them is treated as an independent point. For this purpose, the analyzed geographical area is subdivided into independent cells with related AIS messages. These data points in the grid are analyzed using so-called signature-based or rule-based techniques. The idea of these techniques is the employment of various association rules to detect specific movement changes [7]. Zhu applied database management, data warehouse, and data mining technologies to analyze AIS data [8]. Deng [9] extended the features and inserted time stamps. These extensions enable employing Markov model for supplementation of rules. While declaring the point-based analysis, Pallotta et al. [10] proposed to use a sliding time window to estimate the relationship between successive AIS data points. The obtained waypoints are clustered using Density-Based Spatial Clustering of Applications with Noise methodology and employed for anomaly detection and movement prediction. Despite the claims about point-based analysis, the authors implemented the idea of updating the traffic knowledge from the input of AIS messages and the use of historical knowledge. The same clustering methodology was explored in [11]. Here, the historical spatiotemporal data are analyzed to detect waypoints of routes.

The main weakness of point-based techniques is the analysis of movement short-term history or disregard of history even. The planned and purposing vessel movement should generate highly-correlated AIS data, and this can be used for movement anomaly detection. On the other hand, a limited number of analyzed data points means real-time calculation and decision making. This quality makes point-based anomaly detection techniques attractive for real-time tasks. Nevertheless, at the moment, the prevalence of these techniques is quite limited.

Trajectory-based techniques treat the entire traffic data sequence as a whole. Several research directions are analyzed in the literature related to the analysis of vessel trajectories: maritime traffic pattern mining, ship collision risk assessment [12], maritime anomaly detection [13,14,15], identification of the types of ships [16], and combating abalone poaching [17].

In the case of trajectory-based detection, models of normal movement are created (using the entire trajectory data, not part of it) and the anomalies are detected as movement data inadequacy to the model. Thus, these techniques are characterized by having a huge amount of AIS data to analyze. This property requires some data pre-processing such as compression or clustering.

In [18], a piece-wise linear segmentation is applied to compress the data of vessel trajectories, and then the similarity of trajectories (for detection of anomalies) is performed using alignment kernels (dynamic time warping and edit distances, namely). The model by Lei [13] defines spatial, sequential, and behavioral features of the vessel movement. The movement anomaly is detected as the outlying features of the trajectory model, and the degree of suspiciousness is evaluated. The geometrical properties of the trajectory are employed in [19]. Here, the vessel trajectory is compared with the graph search-based path and the difference is estimated by a final score. The threshold value of the score is employed as the decision and labeling value. Another trajectory-based analysis techniques can be found in [20,21,22].

Analysis of the entire trajectory gives the advantage of the historical movement data, which can be essential for anomaly detection. However, full data analysis requires much more complicated algorithms such as neural networks. This complicates the application of trajectory-based analysis for real-time tasks. In addition, such algorithms are sensitive to missing data (e.g., lost AIS messages).

A comprehensive and categorizing review on maritime anomaly detection can be found in [5,15,23].

Analysis of full trajectory data and anomaly detection would require data-driven approaches such as artificial neural network-based or statistical methods. These approaches can perform in an unsupervised or semi-supervised manner (i.e., they do not need labeled data) and can cope with large amounts of data. The issue of real-time calculations should be solved using the idea of incremental modeling (retraining, re-estimating, etc.): the model of vessel movement should be updated concerning recent data to avoid of complete remodeling or model retraining.

## 3. Motivation

The vessel movement (normal or abnormal) can be treated differently regarding the sea region where the movement is observed. For example, if the ship is quite distant from the seaport, then even high decline from its course cannot be indicated as an anomaly: weather condition, stormy sea, etc. may have a great influence on vessel trajectory. On the other hand, if vessel movement is observed at the seaport surroundings, even a small deviation from the course may be thought as abnormal vessel activity. To this purpose, the method used for traffic anomaly detection has to have a feature that allows different region scaling at different maritime traffic observation areas. The self-organizing map (SOM) method has such a scaling property. SOM is a neural network-based method that is trained in an unsupervised way using a competitive learning [24,25,26,27]. The neural network can be used for both visualization and clustering of multidimensional data [28].

In the previous research [29], the modified SOM algorithm for maritime vessel movement data classification into normal and abnormal classes is presented. The modification is achieved by incorporating virtual pheromone intensity calculations at the last epoch of model training. During the model validation stage, the pheromone intensity threshold is established by applying a gradient descent method. The dependence of the network neighboring function on the classification results was investigated; the best classification accuracy qA achieved using the Mexican hat neighboring function. The influence of different SOM grid dimensions on the classification results of the proposed algorithm has been investigated. It was proved experimentally that the algorithm achieved the best precision using grid dimension 25 × 25. This knowledge was used as a starting point for the network data batching and training strategies investigation presented in this paper.

With the growth of maritime traffic, especially near seaports, the complete retraining of the SOM algorithm becomes costly in terms of training time. The need for algorithm retrain quite straightforward: the more vessel movement data that are observed and fed into the algorithm, the better the precision of the algorithm should be. All neural networks are strongly dependent on the input sequence in the training data. It was observed that, if only the input sequence of the data changes, even though the system architecture stays the same, classification accuracy results may be significantly impaired [30]. Other authors proposed neural networks retraining strategies to build compact neural network models with less memory usage and faster inference speed [31]. Recently, the SOM neural network is being used to build datasets used in deep neural network model retraining [30,32] or is used as a part of deep neural network model [33]. Different areas of applications of the SOM algorithm depicts the necessity to investigate more thoroughly algorithm effectiveness with respect to algorithm sensitivity, precision and data processing time by introducing different retraining strategies. SOM retraining ensures the inclusion of the most recent movement data that reflects actual conditions and context. To maintain high algorithm precision and sensitivity, approaches to data streaming, batching and model retrain strategies has to be explored [34]. In this article, the authors introduce two neural network retrain strategies and compare the results with the standard procedure of neural network model experimental investigation (so-called Strategy I).

- Strategy I presents data batching and algorithm training whenever the new batch becomes available as if no model history data were available. It is a common approach for neural network training/validation/testing. In this paper, it is used as a reference with the view to compare retrain Strategies II and III introduced by the authors.
- Strategy II presents algorithm performance while using pre-trained model parameters on previously trained data with the newly arriving data batches.
- Strategy III presents different data batch shuffling techniques and the use of previously pre-trained model parameters.

All three strategies investigate the learning rate parameter influence on the model performance and training time as well. Data passed from a vessel can be viewed as a stream that contains facts regarding vessel movement trajectories. Those may depend on seasonal data, the shipping routes, schedules, and so on. Thus, the abnormality detection model has to be developed by analyzing vessel movement trajectories (as well as historical data) in an incremental manned based on the up-to-date data it receives.

## 4. Experiments

In this section, we present a detailed description of the SOM network retraining strategies and results of the experiments using real datasets.

### 4.1. Data Preparation

The detailed description of the previous study of SOM size and modification by introducing the SOM evaporation functions are presented in [29]. Data from the region of medium maritime traffic at the Klaipeda seaport were selected for the analysis of the proposed retraining strategies of the SOM network. During the experiments, two datasets were used: Cargo vessels and Passenger vessels. Each item (point) of a vessel’s streamed data is described by longitude, latitude, heading, vessel speed, wind direction, wind speed, wave direction, and wave height values. The Cargo dataset is represented by 180,300 and the Passenger dataset is described by 43,879 vessel movement observation items that were registered in a streamed manner. All experiments in this section were carried out with the Cargo dataset; afterwards, the data batching strategies were tested on the Passenger dataset.

**Figure 1:**
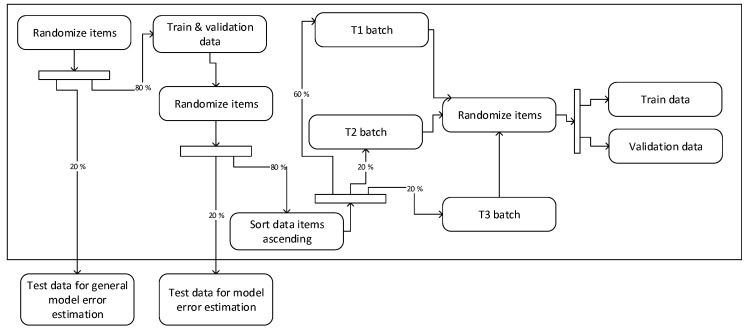
Data split scheme.

First, 20% of the Cargo vessel dataset was randomly selected for the general model error evaluation. Then, the resulting 80% of the dataset items were used for the data batching strategy investigation. These 80% of data items were split into 20% for strategy testing, and 80% for T1, T2, and T3 data batch splitting (see Figure 1) to perform the SOM network training and validation. Batches were used in the experiments to imitate the continuous data arrival with the view to investigate different SOM network retraining strategies and learning rate parameter selection. The scheme of data split is shown in Figure 1.

All data items were sorted in ascending order with respect to data sending time. The SOM network of size 25 × 25 was taken according to the SOM size investigation published in [29].

### 4.2. Training Strategies of the SOM Network

Strategy I. For the SOM network training and validation, we used T1, T2 and T3 data batches. The learning rate parameter was set to 0.5. Then, after the network was trained and validated with the T1 data batch, the new data were fed to the network as follows: the T1 and T2 batch data were merged together and the algorithm was trained from the initial random state using all items from T1 and T2. The same scheme was applied to the T3 data batch.

To get the best network performance, the learning rate parameter can be adjusted. Initial research led us to divide the learning rate parameter search into these intervals and step sizes: in the interval [0.005;0.04], step was set to 0.005; in the interval [0.04;0.1], step size was increased to 0.01; and, in the interval [0.1;0.5], step size was set to 0.1 (see Table 1). In this way, the training experiment of Strategy I was repeated while every learning parameter value was tested to achieve the best algorithm performance. After the model was trained, it was tested with the test dataset, which allowed evaluating the general model error. The best-obtained model characteristics with model test dataset are presented in Table 1 (bold line).

The statistics of the best Strategy I model using test data for general model error estimation and test data for model error estimation is presented in Table 2. The time needed for the algorithm retraining was 40,769 s. Strategy II. The initial algorithm was trained 10 times with the T1 batch data. During each training, the weights of the SOM network were generated randomly, and the best performing network was selected while keeping a fixed learning rate parameter at the value of 0.5. The performance of the investigated network on repetitive Strategy II (using only T1 dataset) model evaluation and testing is presented in Table 3. The line marked in bold shows the best network obtained. Quite small deviations of the precision and the sensitivity rates show the network stability. Then, the best-obtained network parameters were used as initial weights for the network to be trained with T2 batch data. Finally, imitating the new data portion arrival, the best model obtained with T2 batch data was retrained with the T3 batch data. The results of the additional experiment show that the best performance network was obtained with learning rate 0.025.

The statistics (model test error and general model error evaluation) of the best model data are presented in Table 4. The time needed for model training was 18,229 s.

Strategy III. The scheme of the model training validation and testing was similar to that described in Strategy II, except for the following two things. Firstly, from T2 and T3 batches, there were produced four data batches (Tm2–Tm5), each containing one quarter of both T2 and T3 data (see Table 5). Secondly, as previously described, after every model training and validation, the parameters of the best-obtained model were used for every next Tm2–Tm5 batch training, except the model training data aggregation. For every retraining. test data for model error estimation of data was used as described in previous Strategies I and II. Half the items from Tm2–Tm5 data batches were compounded of items from T2 and T3, as shown in Table 5 (Tm2–Tm5) while another part of the data was selected proportionally, with respect to those data points attached to the previous best model SOM winning neurons. This approach guaranteed that the knowledge of frequently passed sea regions was incorporated into the next model training because it is not frequent for the ships to change their sea routes. Experiments depicted that the best model was obtained with the learning rate being 0.03.

The statistics of the Strategy III best model were obtained using test data for general model error estimation, and the results are presented in Table 6.

The time needed for the algorithm retraining was 27,854 s. The summary of relative time required for the training Strategies I–III is presented in Table 7.

The same data batching Strategies I–III described above were tested on the Passenger dataset as well. The results are presented in Table 8.

From the results shown in Table 7 and Table 8, it can be seen that, by applying different SOM model retraining Strategies, while keeping the same data batch sizes, it is possible to substantially decrease the time for maritime traffic abnormal movement detection while retraining the model precision and sensitivity at very high values. The results obtained show that the SOM network could be retrained in half the time while keeping precision and sensitivity at almost the same high values. The results presented in Table 8 prove the correctness of the training strategies investigation.

## 5. Conclusions

This paper extends the previous study on a self-organizing map application, which is trained in an unsupervised way using competitive learning, for processing of sensors stream data in order to detect abnormal vessel movement in maritime traffic. Different strategies for the unsupervised retraining of the SOM network to classify maritime vessel movement data into normal and abnormal classes were presented and investigated. The data batching strategies ensure high precision of the algorithm by introducing a huge amount of new data on vessel movements. Two different unsupervised SOM network retraining strategies for maritime vessel movement data classification into normal and abnormal classes were proposed and investigated. The experimental research depicted promising results. The study showed that the SOM network can be retrained in half the time by only applying different train/validation and test datasets. The initial results depict that the obtained speed-up in data processing time maintains precision and sensitivity, varying not more than 3% in unusual maritime traffic detection.

The results of the experiments show that:

- If the model is trained from initial random weights of the SOM network, the best performance is observed; however, the training time is the longest. Model precision reaches 0.979 and sensitivity 0.889 at learning rate 0.5.
- If the model is trained on top of the pre-trained model weights, the precision and sensitivity slightly drop, but the training time decreases by half at learning rate 0.025.
- If the model is trained on top of the pre-trained model weights and the newly arrived data batch is proportionally mixed with those winning neurons, training time can be decreased by one third while keeping almost the same results as depicted previously at learning rate 0.03.

The independent experiment on unseen dataset confirmed the results correctness and allowed concluding that, by applying batched data approach for SOM retraining on the pre-trained model, network training can be shortened to half the time by selecting learning rate parameter from the interval [0.025;0.03] while maintaining the model sensitivity and precision with only minor changes.

## Figures and Tables

**Table 1:** Selection of learning rate.

Learning Rate	TP	FP	TN	FN	Precision	Sensitivity
0.005	924	519	26,648	757	0.6403	0.5497
0.010	943	505	26,662	738	0.6512	0.5610
0.015	957	498	26,669	724	0.6577	0.5693
0.020	963	487	26,680	718	0.6641	0.5729
0.025	968	478	26,689	713	0.6694	0.5758
0.030	976	471	26,696	705	0.6745	0.5806
0.035	986	468	26,699	695	0.6781	0.5866
0.040	998	461	26,706	683	0.6840	0.5937
0.050	1025	445	26,722	656	0.6973	0.6098
0.060	1066	413	26,754	615	0.7208	0.6341
0.070	1109	394	26,773	572	0.7379	0.6597
0.100	1197	303	26,864	484	0.7980	0.7121
0.200	1431	135	27,032	250	0.9138	0.8513
0.300	1486	81	27,086	195	0.9483	0.8840
0.400	1500	55	27,112	181	0.9646	0.8923
**0.500**	**1510**	**52**	**27,115**	**171**	**0.9667**	**0.8983**
0.600	1507	54	27,113	174	0.9654	0.8965
0.700	1502	59	27,108	179	0.9622	0.8935

**Table 2:** Training Strategy I performance at learning rate 0.5.

Stage	TP	FP	TN	FN	Precision	Sensitivity
Testing (model error)	1510	52	27,115	171	0.9667	0.8983
Testing (general error)	1868	69	33,890	233	0.9644	0.8891

**Table 3:** Strategy II performance on model test data.

No.	TP	FP	TN	FN	Precision	Sensitivity
1	1364	241	26,926	317	0.8498	0.8114
2	1329	280	26,887	352	0.8260	0.7906
3	1359	252	26,915	322	0.8436	0.8084
4	1364	274	26,893	317	0.8327	0.8114
5	1356	253	26,914	325	0.8428	0.8067
6	1335	253	26,914	346	0.8407	0.7942
7	1314	251	26,916	367	0.8396	0.7817
8	1332	258	26,909	349	0.8377	0.7924
**9**	**1367**	**237**	**26,930**	**314**	**0.8522**	**0.8132**
10	1338	240	26,927	343	0.8497	0.7960
				max	0.8522	0.8132
				min	0.8260	0.7817
				average	0.8413	0.8011
				stdev	0.0079	0.0115

**Table 4:** Retraining Strategy II performance at learning rate 0.025.

Stage	TP	FP	TN	FN	Precision	Sensitivity
Testing (model error)	1500	98	27,069	181	0.9387	0.8923
Testing (general error)	1836	122	33,837	265	0.9377	0.8739

**Table 5:** Partitioning of dataset (Strategy III).

Data Batches	% of Train and Validation Data	New Data Items	All Data Items
T1	60%	69,235	69,235
Tm2	10%	11,539	23,078
Tm3	10%	11,539	23,078
Tm4	10%	11,539	23,078
Tm5	10%	11,539	23,078

**Table 6:** Retraining Strategy III performance at learning rate 0.003.

Stage	TP	FP	TN	FN	Precision	Sensitivity
Testing (model error)	1527	73	27,094	154	0.9544	0.9084
Testing (general error)	1866	91	33,868	235	0.9535	0.8881

**Table 7:** Retraining Strategies I–III performance on Cargo dataset.

Strategy	Precision	Sensitivity	Relative Time
Strategy I	0.9644	0.8891	1
Strategy II	0.9377	0.8739	0.4471
Strategy III	0.9535	0.8881	0.6832

**Table 8:** Retraining Strategies I–III performance on Passenger dataset.

Strategy	Precision	Sensitivity	Relative Time
Strategy I	0.9795	0.8897	1
Strategy II	0.9802	0.8870	0.4478
Strategy III	0.9817	0.8888	0.6817
